# Síncope como Expressão Fenotípica da Amiloidose Hereditária por Transtirretina Val142Ile (Val122Ile)

**DOI:** 10.36660/abc.20180130

**Published:** 2020-05-11

**Authors:** Nágela S. V. Nunes, João Paulo Moreira Carvalho, Fernanda Salomão Costa, Marcelo Souto Nacif, Joelma Dominato, Claudio Tinoco Mesquita, Evandro Tinoco Mesquita

**Affiliations:** 1 Complexo Hospitalar de Niterói NiteróiRJ Brasil Complexo Hospitalar de Niterói – Cardiologia, Niterói, RJ – Brasil; 2 Hospital Universitário Antônio Pedro Departamento de Cardiologia (Ebserh/UFF) NiteróiRJ Brasil Hospital Universitário Antônio Pedro, Departamento de Cardiologia (Ebserh/UFF), Niterói, RJ – Brasil; 3 Labs a+ / Grupo Fleury RJ NiteróiRJ Brasil Labs a+ / Grupo Fleury RJ – Ecocardiografia, Niterói, RJ – Brasil; 4 Hospital Pró-Cardíaco Rio de JaneiroRJ Brasil Hospital Pró-Cardíaco - Medicina Nuclear, Rio de Janeiro, RJ – Brasil; 5 Hospital Universitário Antonio Pedro Departamento de Radiologia (Ebserh/UFF) NiteroiRio de Janeiro Brasil Hospital Universitário Antonio Pedro, Departamento de Radiologia (Ebserh/UFF), Niteroi, Rio de Janeiro – Brasil; 6 Americas Medical City Centro de Educação e Treinamento Edson Bueno Rio de JaneiroRJ Brasil Americas Medical City - Centro de Educação e Treinamento Edson Bueno, Rio de Janeiro, RJ – Brasil

**Keywords:** Síncope, Amiloidose Familiar/genética, Neuropatias Amiloides Familiares, Cardiomiopatias/diagnóstico, Diagnóstico por Imagem/métodos, Prevalência

## Introdução

A amiloidose por transtirretina (ATTR) é uma doença familiar causada por uma das mais de 100 mutações descritas, em que há produção de amiloides que se depositam nos tecidos.^1^ As fenocópias abrangem neuropatia (autonômica e periférica), cardiomiopatia, acometimento renal, gastrointestinal, de vítreo e de meninge, que variam de acordo com a mutação genética, etnia e origem geográfica, mesmo entre indivíduos com a mesma mutação ou dentro da mesma família.^2^

A síncope (perda transitória da consciência causada por hipoperfusão cerebral global) na presença de cardiopatia, confere risco de eventos fatais.^3^ A mutação Val142Ile tem a insuficiência cardíaca com fração de ejeção preservada (ICFEP) como fenótipo clínico predominante, sendo a síncope um sintoma pouco comum.^4^^,^^5^

## Relato do Caso

Homem, 64 anos, branco, engenheiro, natural do Rio de Janeiro. Relatava episódio isolado de síncope ao levantar-se rápido da posição sentada, após corrida. Histórico de morte súbita na família (tio aos 60 anos). Usava escitalopram 10 mg/dia e finasterida 5 mg/dia.

Exame físico: IMC de 21,8 Kg/m^2^ e turgência jugular a 45°. PA: 140x80 mmHg, FC: 85 bpm, FR: 18 ipm, quarta bulha, ictus sustido e palpável no 6º espaço intercostal na linha hemiclavicular, pulmões limpos e edema de tornozelos. Vinha em CF I.

Sangue: peptídeo natriurético do tipo B (BNP): 233 pg/ml (VN: até 100 pg/ml) e Troponina US: 0,135 ng/ml (VN: até 0,01 ng/ml).

**Figura 1:**
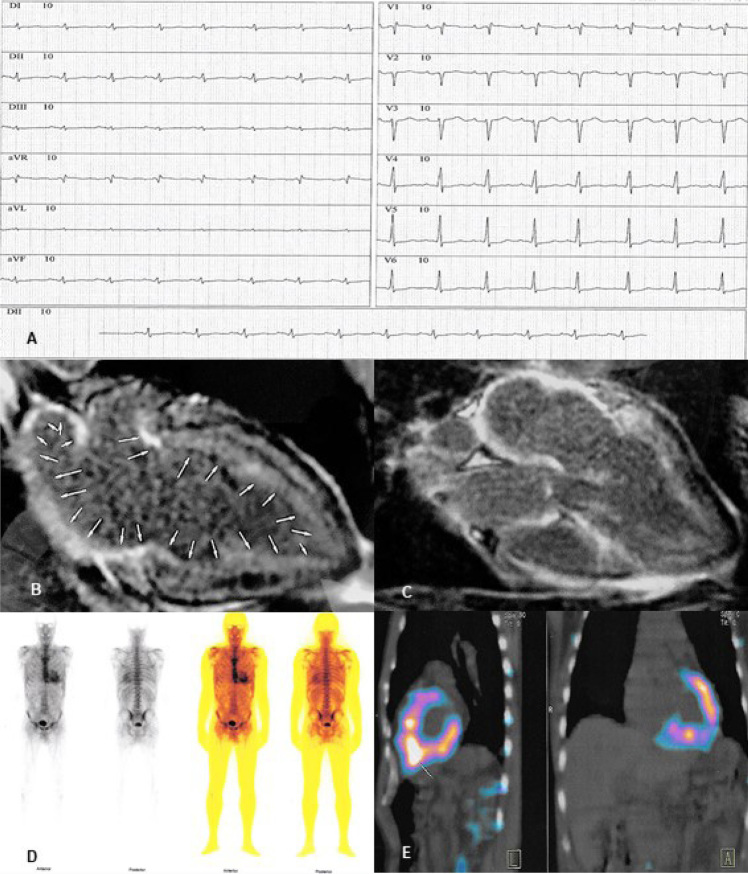
A) Eletrocardiograma: Ritmo sinusal, FC: 88 bpm, eixo do QRS indeterminado. Onda P no plano frontal com duração aumentada (160 ms), com bloqueio parcial do feixe de Bachmann e onda P tricuspídea em D2, D3 e aVF; e no plano horizontal observa-se padrão qR em V1 e índice de Morris, o que traduz aumento dos átrios direito e esquerdo. Observa-se também amputação de R anterosseptal e presença de baixa voltagem no plano frontal. B e C) Ressonância magnética do coração em repouso mostra HVE difusa, com áreas de realce tardio subendocárdico difuso no VE (setas), nos átrios e no septo interatrial. D e E) Cintilografia miocárdica com Pirofosfato de 99m-Tecnécio mostrando intensa fixação do radiotraçador no miocárdio (escore grau 3).

Eletrocardiograma (ECG) (Figura 1): ritmo sinusal, FC: 84 bpm, bloqueio de ramo direito, baixa voltagem nas derivações frontais e amputação de R anterosseptal.

**Figura 2:**
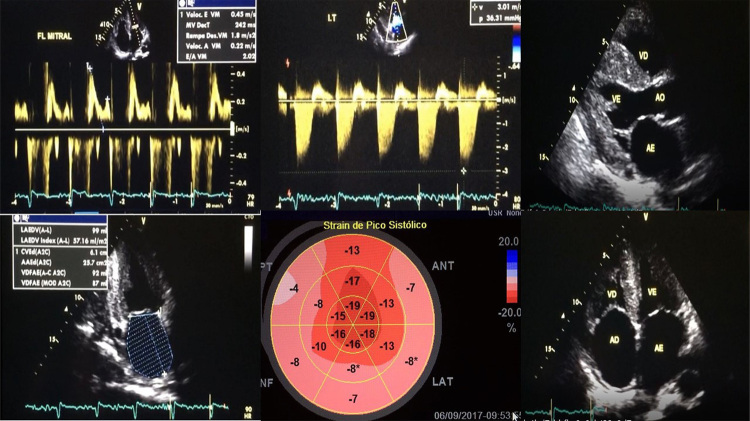
Ecocardiograma transtorácico mostrando padrão do Doppler mitral do tipo pseudonormal (disfunção diastólica tipo II), pico de velocidade da insuficiência tricúspide > 2,8 m/s e aumento biatrial com volume indexado do átrio esquerdo de 87 ml/m2. Cortes longitudinal paraesternal e apical 4 câmaras mostrando aumento do brilho miocárdico, espessamento do septo interatrial e das valvas e HVE. Strain global longitudinal com padrão típico de amiloidose - "relativa preservação das regiões apicais" e redução do strain longitudinal nos segmentos basal e médio do miocárdio.

Ecocardiograma (ECO TT): hipertrofia ventricular esquerda (HVE) - septo = 16 mm e parede posterior = 13 mm - fluxo mitral com déficit de relaxamento tipo II e volume indexado do átrio esquerdo: 87 ml/m^2^ (Figura 2). Holter de 24 h e teste ergométrico apresentavam surtos curtos e assintomáticos de taquicardia ventricular polimórfica (TVP).

Solicitada ressonância magnética do coração (RMC) em repouso e após estresse com dipiridamol, que mostrou HVE difusa e ausência de isquemia miocárdica, com áreas de realce tardio (RT) mesocárdico lateral e anterior e subendocárdico difuso no ventrículo esquerdo (VE), nos átrios e no septo interatrial (Figura 1). Avaliação do *strain* global longitudinal (SGL), após RMC, mostrou alterações marcadas nas porções basal e medial de todas as paredes do miocárdio, poupando as regiões apicais do VE, o que era compatível com padrão descrito de amiloidose cardíaca (AC).

A biópsia de gordura abdominal e reto confirmaram o diagnóstico de amiloidose, pela coloração vermelho congo. Imunofixação no sangue e na urina de 24 horas e dosagem de cadeias leves no sangue afastaram gamopatia monoclonal, sendo solicitada, após isso, cintilografia miocárdica (CM) Pirofosfato de 99m-Tecnécio (Figura 1), que mostrou intensa fixação do radiotraçador no miocárdio (escore grau 3), sugerindo ser a etiologia da AC do tipo ATTR.

Por fim, o paciente foi submetido a teste genético, o qual confirmou mutação em heterozigose para o gene da transtirretina (TTR) do tipo Val142Ile.

## Discussão

Quando se avalia um paciente vítima de síncope, é prioritária a estratificação do risco de eventos fatais,^3^ que levam em conta alterações eletro e ecocardiográficas, as quais estavam presentes neste paciente. As alterações encontradas no ECG (Figura 1), na presença de HVE, já são sinais de alerta para o diagnóstico de AC.^1^^,^^4^^,^^6^

O BNP e a troponina elevados traduziam aumento de pressões intracavitárias e injúria miocárdica em curso, o que era indicativo de doença cardíaca.

O ECO TT confirmou a suspeita de cardiopatia. As alterações encontradas (Figura 2), a história familiar de morte súbita e TVP ao esforço, levantaram suspeita de síncope cardíaca e os diagnósticos diferenciais principais seriam miocardiopatia hipertrófica (MCPH), doença arterial coronariana (DAC) e AC.^3^

A RMC, solicitada a seguir, forneceu evidências fortemente sugestivas de AC, dado ao padrão característico do RT, afastando as hipóteses de MCPH e de DAC. Por ser o gadolíneo um agente puramente extracelular e não penetrar no cardiomiócito intacto, a aparência característica do RT (Figura 1) em território não coronariano é extremamente sugestiva de AC e foi determinante no caso relatado.^1^^,^^4^^,^^7^

A avaliação da deformidade miocárdica pela técnica do SGL, realizada após a RMC, demonstrou um padrão típico de AC (Figura 2), o que pôde descartar outras causas de HVE e corroborar o diagnóstico, o que tem sido muito útil neste cenário.^8^

Dentre os tipos de AC, a causada por cadeias leves de imunoglobulina (AL) é a que mais comumente acomete o coração, por isso, inicia-se a investigação pela busca de doença hematológica.^1^^,^^4^^,^^6^ Como o diagnóstico definitivo de AC requeria, à época, biópsia tissular, assim foi feito. Algoritmos diagnósticos mais recentes reservam a biópsia tissular apenas para os casos suspeitos de AL, já que a CM com Pirofosfato de 99m-Tecnécio substitui a biópsia miocárdica na ATTR.^1^^,^^9^

Essa técnica é usada há tempos para diagnóstico de patologias ósseas, cujo radiotraçador tem grande afinidade pelo cálcio, o qual está presente, quase sempre, apenas nos depósitos da ATTR. Os valores preditivos positivos e negativos para o diagnóstico de ATTR pela CM com escore ≥ 2 é de 88 e 100% respectivamente. Propõe-se certeza diagnóstica quando há escore ≥ 2 na ausência de pico monoclonal de imunoglobulina, o que equivaleria à biópsia endomiocárdica,^9^ como aconteceu no caso descrito (Figura 1), confirmado pelo teste genético.

Os sintomas cardiológicos mais frequentes na ATTR Val142Ile são: insuficiência cardíaca, dispneia, arritmias e tonteira. Síncope é um achado incomum (8%), mais frequente AL (20%), e quando acontece no esforço representa a inabilidade em aumentar o débito cardíaco, o que confere alta mortalidade.^5^^,^^10^ Além disso contribuem para a ocorrência de síncope, a sensibilidade a depleção de fluidos do intravascular combinada a neuropatia autonômica, reserva miocárdica deprimida, disfunção e rigidez atrial e a presença de arritmias.^6^ Todas essas possibilidades fazem da síncope uma apresentação multifatorial na AC, como pode ter ocorrido no caso descrito.

O THAOS, um registro mundial, aberto a todos os pacientes portadores de ATTR, mostra que a mutação Val142Ile (também conhecida como Val122Ile) é a segunda mutação mais comum no mundo e a mais comum nos EUA, contabilizando 23% do total neste país e 1% do resto do mundo. Os portadores dessa mutação são, em sua maioria, afrodescendentes e homens, sendo prevalente em 3 a 4% de afroamericanos ao nascerem, com penetrância de aproximadamente 20%.^5^

A ATTR é uma causa subdiagnosticada de ICFEP, embora depósitos de TTR sejam identificados em até 30% de idosos encaminhados a autópsia.^1^^,^^2^^,^^5^ Síncope apesar de incomum na apresentação desse fenótipo, pode ser o primeiro sintoma dessa enfermidade.

## References

[1] 1. Nativi-Nicolau J, Maurer MS. Amyloidosis cardiomyopathy. Curr Opin Cardiol. 2018;33(5):571–9. 10.1097/HCO.0000000000000547 30015648

[2] 2. Ando Y, Coelho T, Berk JL, Waddington Cruz M, Ericzon B-G, Ikeda S-I, et al. Guideline of transthyretin-related hereditary amyloidosis for clinicians. Orphanet J Rare Dis. 2013 Feb 20;8:31. 10.1186/1750-1172-8-31 PMC3584981 23425518

[3] 3. Brignole M, Moya A, de Lange FJ, Deharo J-C, Elliott PM, Fanciulli A, et al. 2018 ESC Guidelines for the diagnosis and management of syncope. Eur Heart J. 2018;39(21):1883–948. 10.1093/eurheartj/ehy037 29562304

[4] 4. Elliott P. Addressing common questions encountered in the diagnosis and management of cardiac amyloidosis. Circulation. 2017;135(14):1357–77. 10.1161/CIRCULATIONAHA.116.024438 PMC5392416 28373528

[5] 5. Maurer MS, Hanna M, Grogan M, Dispenzieri A, Witteles R, Drachman B, et al. Genotype and Phenotype of Transthyretin Cardiac Amyloidosis. J Am Coll Cardiol. 2016;68(2):161–72. 10.1016/j.jacc.2016.03.596 PMC4940135 27386769

[6] 6. Banypersad SM, Moon JC, Whelan C, Hawkins PN, Wechalekar AD. Updates in Cardiac Amyloidosis: A Review. J Am Heart Assoc. 2012;(2):1–13. 10.1161/JAHA.111.000364 PMC3487372 23130126

[7] 7. Martinez-Naharro A, Treibel TA, Abdel-Gadir A, Bulluck H, Zumbo G, Knight DS, et al. Magnetic Resonance in Transthyretin Cardiac Amyloidosis. J Am Coll Cardiol. 2017;70(4):466–77. 10.1016/j.jacc.2017.05.053 28728692

[8] 8. Phelan D, Thavendiranathan P, Popovic Z, Collier P, Griffin B, Thomas JD, et al. Application of a Parametric Display of Two-Dimensional Speckle-Tracking Longitudinal Strain to Improve the Etiologic Diagnosis of Mild to Moderate Left Ventricular Hypertrophy. J Am Soc Echocardiogr. 2014; 27(8):888–95. 10.1016/j.echo.2014.04.015 24874973

[9] 9. Gillmore JD, Maurer MS, Falk RH, Merlini G, Damy T, Dispenzieri A, et al. Nonbiopsy diagnosis of cardiac transthyretin amyloidosis. Circulation. 2016;133(24):2404–12. 10.1161/CIRCULATIONAHA.116.021612 27143678

[10] 10. Marin-Acevedo JA, Sanchez-Alvarez C, Alsaad AA, Pagán RJ. Case Report Recurrent syncope, a clue in amyloid cardiomyopathy. Case Rep Med. 2018 Jan 28:1-6. 10.1155/2018/1864962 PMC5829326 29559999

