# Supplementary material for: Síncope como Expressão Fenotípica da Amiloidose Hereditária por Transtirretina Val142Ile (Val122Ile)
Source: Arq Bras Cardiol. 2020 May 11;114(4 Suppl 1):1–3. [Article in Portuguese] doi: 10.36660/abc.20180130 (PMC8149112; doi:10.36660/abc.20180130)
Supplement: Translation

# Translation: Syncope as a Phenotypic Expression of Hereditary Transthyretin Amyloidosis Val142Ile (Val122Ile)

**Authors:** Nágela S. V. Nunes, João Paulo Moreira Carvalho, Fernanda Salomão Costa, Marcelo Souto Nacif, Joelma Dominato, Claudio Tinoco Mesquita, Evandro Tinoco Mesquita

**Affiliations:** 1 NiteróiRJ Brazil Complexo Hospitalar de Niterói – Cardiologia, Niterói, RJ – Brazil; 2 NiteróiRJ Brazil Hospital Universitário Antônio Pedro, Departamento de Cardiologia (Ebserh/UFF), Niterói, RJ – Brazil; 3 NiteróiRJ Brazil Labs a+ / Grupo Fleury RJ – Ecocardiografia, Niterói, RJ – Brazil; 4 Rio de JaneiroRJ Brazil Hospital Pró-Cardíaco - Medicina Nuclear, Rio de Janeiro, RJ – Brazil; 5 NiteroiRio de Janeiro Brazil Hospital Universitário Antonio Pedro, Departamento de Radiologia (Ebserh/UFF), Niteroi, Rio de Janeiro – Brazil; 6 Rio de JaneiroRJ Brazil Americas Medical City - Centro de Educação e Treinamento Edson Bueno, Rio de Janeiro, RJ – Brazil

**Keywords:** Syncope, Amyloidosis, Familial/genetics, Amyloid Neuropathies, Familial, Cardiomyopathies/diagnosis, Diagnostic, Imaging/methods, Prevalence

## Introduction

Transthyretin amyloidosis (ATTR) is a familial disease caused by one of more than 100 described mutations, where there is production of amyloids that are deposited in tissues.^1^ Phenocopies include neuropathy (autonomic and peripheral), cardiomyopathy, renal, gastrointestinal, vitreous and meningeal involvement, which vary according to the genetic mutation, ethnicity and geographical origin, even among individuals with the same mutation or within the same family.^2^

Syncope (transient loss of consciousness caused by global cerebral hypoperfusion) in the presence of heart disease confers risk of fatal events.^3^ The Val142Ile mutation has heart failure with preserved ejection fraction (HFpEF) as the predominant clinical phenotype, with syncope being an uncommon symptom.^4^^,^^5^

## Case Report

The patient was male, 64 years old, of white ethnicity, engineer, born in Rio de Janeiro. He reported an isolated episode of syncope when he quickly got up from a sitting position after running. There was a history of sudden death in the family (uncle at 60). He used to take escitalopram 10 mg/day and finasteride 5 mg/day.

At physical examination: BMI of 21.8 kg/m^2^ and jugular turgency at 45o. BP: 140x80 mmHg, HR: 85 bpm, RR: 18 breaths per minute, fourth heart sound, sustained and palpable apex beat in the 6o intercostal space at the hemiclavicular line, clear lungs and ankle edema. He was in NYHA FC I. Blood: BNP: 233 pg/mL (NV: up to 100 pg/mL) and ultra-sensitive Troponin: 0.135 ng/mL (NV: up to 0.01 ng/mL).

The electrocardiogram (EKG) (Fig.1) showed sinus rhythm, HR: 84bpm, right bundle branch block, low voltage in frontal leads, and an pseudo-infarct pattern in precordial leads.

**Figure 1:** A) Electrocardiogram: Sinus rhythm, HR: 88 bpm, indeterminate QRS axis. P-wave in the frontal plane with increased duration (160 ms), with partial Bachmann's bundle block and tricuspid P wave in D2, D3 and aVF; qR pattern in V1 and Morris index are observed in the horizontal plane, which means right and left atrial enlargement. Pseudo-infarct pattern in precordial leads and presence of low voltage in the frontal plane are also observed. B and C) Resting Cardiac MRI shows diffuse LVH, with areas of diffuse subendocardial late enhancement in the LV (arrows), atria, and interatrial septum. D and E) 99mTc-PYP myocardial scintigraphy showing intense radiotracer uptake in the myocardium (Grade 3).

The transthoracic Echocardiogram (TTE) showed left ventricular hypertrophy (LVH) - septum = 16 mm and posterior wall = 13 mm - mitral flow with type II relaxation deficit and LA indexed volume: 87 mL/m^2^ (Figure 2). 24-h Holter and Exercise Test (ET) showed short and asymptomatic polymorphic ventricular tachycardia (PVT) outbreaks.

**Figure 2:** Transthoracic echocardiogram showing pseudonormal mitral Doppler pattern (type II diastolic dysfunction), tricuspid regurgitation peak velocity >2.8 m/s and biatrial enlargement with indexed LA volume of 87 mL/m^2^. Parasternal longitudinal apical-4 chamber projections showing increased myocardial brightness, interatrial septum and valve thickening, and LVH. Global Longitudinal Strain showing typical amyloidosis pattern – “relative preservation of the apical regions” and longitudinal strain reduction in the basal and middle myocardial segments.

Cardiac magnetic resonance imaging (CMRI) at rest and after stress with dipyridamole requested after TTE showed diffuse LVH and absence of myocardial ischemia, with areas of lateral and anterior mesocardial late enhancement (LE) and diffuse subendocardial LE in the LV, atria and interatrial septum (Figure 1). Evaluation of global longitudinal strain (GLS) after CMRI showed marked alterations in the basal and medial portions of all myocardial walls, sparing the LV apical regions, which was consistent with the described cardiac amyloidosis (CA) pattern.

Abdominal fat and rectal biopsies confirmed the diagnosis of amyloidosis with Congo red staining. Immunofixation in blood and 24-h urine and measurement of light chains Imunoglobulins in the blood ruled out monoclonal gammopathy, after which cardiac technetium-99m pyrophosphate scintigraphy (99mTc-PYP) (Figure 1) was requested, witch showed intense radiotracer uptake in the myocardium (grade 3), suggesting Transthyretin CA (ATTR-CA) etiology.

Finally, the patient underwent genetic testing, which confirmed a heterozygous Val142Ile mutation for the TTR gene.

## Discussion

When evaluating a syncope patient, it is a priority to stratify the risk of fatal events,^3^ which take into account electro and echocardiographic alterations, which were present in this patient. The low voltage pattern found in the EKG (Figure 1), in the presence of LVH, are already warning signs for the diagnosis of CA.^1^^,^^4^^,^^6^

Elevated BNP and troponin levels reflected increased intracavitary pressures and ongoing myocardial injury, which was indicative of heart disease.

The TTE confirmed the suspected heart disease. The (Figure 2), family history of sudden death and the presence of PVT on exertion raised the suspicion of cardiac syncope and the main differential diagnoses would be hypertrophic cardiomyopathy (HCM), coronary artery disease (CAD) and CA.^3^

The CMRI, which was subsequently requested, provided evidence that was strongly suggestive of CA, given the characteristic LE pattern, ruling out the hypotheses of HCM and CAD. Because gadolinium is a purely extracellular agent and does not penetrate the intact cardiomyocyte, the characteristic appearance of LE (Figure 1) in non-coronary territory is extremely suggestive of CA and this was decisive in this case.^1^^,^^4^^,^^7^

The evaluation of myocardial deformity by the GLS technique, performed after the CMRI, demonstrated a typical pattern of CA (Figure 2), which ruled out other causes of LVH and corroborated the diagnosis, which has been very useful in this scenario.^8^

Among the CA types, the one caused by light chains Imunoglobulins (AL) is the one that most commonly affects the heart; therefore, we began with the search for hematological disease.^1^^,^^4^^,^^6^ Since the definitive diagnosis of CA required tissue biopsy at that time, this was performed. More recent diagnostic algorithms reserve tissue biopsy only for suspected cases of AL, as the 99m-Tc-PYP myocardial scintigraphy replaced myocardial biopsy in ATTR-CA.^1^^,^^9^

This technique has been used for a long time to diagnose bone diseases, of which radiotracer has a strong affinity for calcium, that is almost only present in ATTR deposits. Positive and negative predictive values for ATTR diagnosis by 99mTc-PYP myocardial scintigraphy with a score ≥ 2 are 88 and 100%, respectively. Diagnostic certainty is proposed when the score is ≥ 2 in the absence of an immunoglobulin monoclonal peak, which would be equivalent to a positive endomyocardial biopsy,^9^ as in the case described (Figure 1), witch was confirmed latter by the genetic testing.

The most frequent cardiac symptoms in Val142Ile ATTR are: heart failure, dyspnea, arrhythmias and dizziness. Syncope, most frequently found in AL (20%), is unusual in ATTR (8%), and when it occurs on exertion it represents the inability to increase cardiac output, which confers high mortality.^5^^,^^10^ Moreover, sensitivity to intravascular fluid depletion combined with autonomic neuropathy, depressed myocardial reserve, atrial dysfunction and stiffness, and the presence of arrhythmias contribute to the occurrence of syncope.^6^ All these possibilities make syncope a multifactorial presentation in CA, as it may have occurred in the case described herein.

THAOS, an open worldwid registry to all patients with ATTR, shows that the Val142Ile, also known as Val122Ile mutation, is the second most common genotype worldwide and the most common in the USA, accounting for 23% of the total mutations in the country and 1% in the rest of the world. The carriers of this mutation are mostly of African descent and males, being prevalent in 3 to 4% of African-Americans at birth, with a penetrance of approximately 20%.^5^

ATTR is an underdiagnosed cause of HFpEF, although TTR deposits are identified in up to 30% of elderly patients referred for autopsy.^1^^,^^2^^,^^5^ Syncope, although uncommon in the presentation of this phenotype, may be the first symptom of this disease.
